# An Intervertebral Disc (IVD) Regeneration Model Using Human Nucleus Pulposus Cells (iHNPCs) and Annulus Fibrosus Cells (iHAFCs)

**DOI:** 10.1002/adhm.202403742

**Published:** 2025-03-07

**Authors:** Yi Zhu, Qing Liu, Chao Yu, Hui Zhang, Jiamin Zhong, Yonghui Wang, Ou Mei, Ethan Gerhard, Wulin You, Guowei Shen, Changqi Luo, Xingye Wu, Jingjing Li, Yi Shu, Ya Wen, Usman Zeb, Hue H. Luu, Michael J. Lee, Lewis L. Shi, Yang Bi, Jian Yang, Jiaming Fan, Russell R. Reid, Tong‐Chuan He, Liangyuan Wen

**Affiliations:** ^1^ Department of Orthopaedic Surgery The First Affiliated Hospital of Soochow University Orthopaedic Institute Soochow University Suzhou 215006 China; ^2^ Department of Orthopaedic Surgery Beijing Hospital National Center of Gerontology Institute of Geriatric Medicine Chinese Academy of Medical Sciences & Peking Union Medical College Beijing 100005 China; ^3^ Molecular Oncology Laboratory Department of Orthopedic Surgery and Rehabilitation Medicine The University of Chicago Medical Center Chicago IL 60637 USA; ^4^ Department of Orthopaedics Xiangya Hospital of Central South University National Clinical Research Center for Geriatric Disorders Changsha 410017 China; ^5^ Department of Orthopaedic Surgery The Affiliated University‐town Hospital Chongqing Medical University Chongqing 401331 China; ^6^ The Breast Cancer Center Chongqing University Cancer Hospital Chongqing 4000430 China; ^7^ Ministry of Education Key Laboratory of Diagnostic Medicine and Department of Clinical Biochemistry School of Laboratory Medicine Chongqing Medical University Chongqing 400016 China; ^8^ Department of Geriatrics Xinhua Hospital Shanghai Jiao‐Tong University School of Medicine Shanghai 200000 China; ^9^ Department of Orthopedics Jiangxi Hospital of Traditional Chinese Medicine Jiangxi University of Traditional Chinese Medicine Nanchang 330006 China; ^10^ Department of Biomedical Engineering The Pennsylvania State University University Park PA 16802 USA; ^11^ Department of Orthopaedic Surgery Wuxi Hospital Affiliated to Nanjing University of Chinese Medicine Wuxi 214071 China; ^12^ Department of Orthopaedic Surgery BenQ Medical Center The Affiliated BenQ Hospital of Nanjing Medical University Nanjing 210019 China; ^13^ Department of Orthopaedic Surgery Yibin Second People's Hospital Affiliated with West China School of Medicine Yibin 644000 China; ^14^ Department of Gastrointestinal Surgery The First Affiliated Hospital of Chongqing Medical University Chongqing 400016 China; ^15^ Department of Oncology The Affiliated Hospital of Shandong Second Medical University Weifang 261053 China; ^16^ Stem Cell Biology and Therapy Laboratory of the Pediatric Research Institute The National Clinical Research Center for Child Health and Disorders and Ministry of Education Key Laboratory of Child Development and Disorders The Children's Hospital of Chongqing Medical University Chongqing 400016 China; ^17^ School of Biomedical Engineering Capital Medical University Beijing 100069 China; ^18^ Institute of Biotechnology and Genetic Engineering The University of Agriculture Peshawar 25130 Pakistan; ^19^ Research Center for Industries of the Future Department of Materials Science and Engineering School of Engineering Westlake University Hangzhou 310030 China; ^20^ Laboratory of Craniofacial Biology and Development Section of Plastic and Reconstructive Surgery Department of Surgery The University of Chicago Medical Center Chicago IL 60637 USA

**Keywords:** annulus fibrosus cells (AFCs), intervertebral discs, intervertebral disc degeneration, intervertebral disc regeneration, nucleus pulposus cells (NPCs), tissue engineering

## Abstract

Intervertebral disc (IVD) degeneration (IVDD), primarily caused by nucleus pulposus (NP) dehydration, leads to low back pain. While current treatments focus on symptom management or surgical intervention, tissue engineering using IVD‐derived cells, biofactors, and scaffolds offers a promising regenerative approach. Here, human NP cells (NPCs) and annulus fibrosus cells (AFCs) are immortalized with human telomerase reverse transcriptase (hTERT), generating immortalized NPCs (iHNPCs) and AFCs (iHAFCs). These cells express NP and AF‐specific markers, are reversible via FLP recombinase, and are non‐tumorigenic. iHAFCs exhibit osteogenic potential, while iHNPCs show chondrogenic differentiation. A 3D‐printed citrate‐based scaffold was employed to develop an IVD regeneration model, with BMP9‐stimulated iHAFCs in the peripheral region and BMP2‐stimulated iHNPCs in the central region. Histological analysis revealed bone formation in the iHAFC region and cartilage formation in the iHNPC region, mimicking the natural IVD structure. Additionally, an *ex vivo* spine fusion model demonstrated robust bone formation in iHAFC‐treated segments. These findings highlight the potential of iHAFCs and iHNPCs as valuable tools for IVD tissue engineering and regeneration.

## Graphical abstract

This study investigates hTert‐immortalized human nucleus pulposus (iHNPCs) and annulus fibrosus (iHAFCs) cells for intervertebral disc (IVD) regeneration and spinal fusion models. A 3D‐printed citrate‐based scaffold loaded with BMP9‐stimulated iHAFCs and BMP2‐stimulated iHNPCs effectively promotes bone and cartilage formation, mimicking the IVD structure. Additionally, BMP9‐iHAFCs enhance spinal fusion in rat models, demonstrating the potential for ex vivo gene therapy applications.

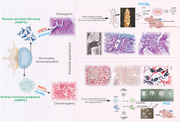

## 1 Introduction

Intervertebral disc (IVD) degeneration‐associated low back pain (LBP) is a chronic and common disorder that affects the middle‐aged and old population worldwide.^[^ ^1^, ^2^, ^3^ ^]^ The IVDs are highly specialized, avascular tissue structures that provide separation and connectivity between vertebrae, act as shock absorbers by counteracting forces that lengthen or compress the spine or affect in a torsional or shear manner, and hold the vertebrae of the spine together while their cartilaginous joints allow for slight mobility in the spine.^[^ ^4^, ^5^ ^]^ A typical IVD consists of different but related tissues including the central highly hydrated nucleus pulposus (NP), the surrounding elastic and fibrous annulus fibrosus (AF), and the cartilaginous endplates (CEP) that connect the vertebral bodies.^[^ ^5^, ^6^ ^]^ NP is a water‐rich tissue with high concentrations of proteoglycans, functioning to maintain the swelling pressure of IVD and absorb shock hydraulicly,^[^ ^7^ ^]^ while the AF has a laminar structure formed by collagen fibers that can resist tensile and compressive strains.^[^ ^2^ ^]^ Thus, the NP distributes hydraulic pressure throughout the intervertebral disc, while the AF encircles the NP as a “cage” to provide structure to the gelatinous form and resist torsion, flexion, and extension movements of the spine.^[^ ^8^ ^]^

Developmentally, vertebrae originate from the sclerotome, and the intervertebral discs form, in part, from the notochord, while notochord degenerates in the regions where the vertebral bodies develop.^[^ ^4^, ^5^, ^8^ ^]^ The remaining notochord expands in the transverse plane to form the gel‐like structure NP, whereas the AF forms from the surrounding ring‐shaped or annular mesenchyme. Within the AF, the inner annulus is initially cartilaginous with a rapid build‐up of type II collagen, while the outer starts with purposefully oriented fibroblastic lamellae that accumulate type I collagen. In addition to water, type II collagen, and proteoglycans, the NP structure contains a low density of cells that produce the extracellular matrix (ECM) products and maintain the integrity of the NP. The ring‐shaped AF consists of highly organized 15 to 25 stacked lamellae of predominantly collagen, with interspersed proteoglycans, glycoproteins, elastic fibers, and connective tissue cells that secrete ECM.^[^ ^5^, ^8^ ^]^

A healthy IVD requires a balance between matrix synthesis and degradation. Such balance can be disturbed, leading to intervertebral disc degeneration (IVDD) which is usually characterized by the loss and dysfunction of IVD cells and the exhaustion of IVD progenitors.^[^ ^5^, ^8^ ^]^ A degenerated IVD usually shows a dehydrated NP with or without disrupted AF, as it is well‐recognized that the IVD degeneration usually originates from the NP region.^[^ ^9^ ^]^ At the early stage of IVDD, conservative treatments are intended to relieve pain and delay the degenerative progress, rather than restore the structure and function of IVD. In patients with late‐stage IVDD, the IVDs usually become decompensated with herniated NP that will compress the spinal cord and cause spinal canal stenosis symptoms such as LBP, limitation of lower limb movement, numbness, incontinence of urine and feces, or even paralysis.^[^ ^10^ ^]^ At this stage, invasive treatments are usually required such as chemonucleolysis, disc replacement, NP discectomy, elastic fixation, and spinal fusion.^[^ ^11^ ^]^

In recent years, tissue engineering by replacing a damaged IVD with scaffolds and appropriate cells has emerged as a potential treatment for IVDD.^[^ ^12^, ^13^, ^14^ ^]^ Successful tissue engineering requires the desirable triad of progenitor cells, biocompatible scaffolds, and factors.^[^ ^12^, ^15^ ^]^ For the past decades, significant advances have been made in developing biocompatible scaffold materials for tissue engineering. Among them, the citrate‐based polymers poly(1,8‐octanediol‐co‐citric acid) (POC) ^16^and polyethylene glycol citrate‐co‐N‐isopropylacrylamide (PPCN)^[^ ^16^ ^]^ have shown great promise as superior biomaterials for regenerative medicine as POC‐based scaffolds are biocompatible and biodegradable.^[^ ^17^ ^]^ A composite of POC with hydroxyapatite has recently been used for manufacturing implantable medical devices cleared for marketing in the USA by the FDA for musculoskeletal surgeries biodegradable, while the thermoresponsive PPCN has been reported for various biomedical applications including the delivery of biologics.^[^ ^17^ ^]^ Another commonly used nontoxic biomaterial hydrogel, Gelatin methacryloyl (GelMA), possesses good mechanical strength, viscoelasticity, and swelling capacity, and promotes chondrogenic differentiation when in high stiffness.^[^ ^18^ ^]^

Even though human IVDs are incapable of self‐repair and spontaneous regeneration, progenitor cells have been identified from NP, AF, and CEP, which raises the hope of reviving degenerated IVDs by differentiating the respective progenitors into corresponding mature IVD cells to promote IVD matrix production and fulfill IVD functions.^[^ ^8^ ^]^ Thus, a profound understanding of the cellular and molecular characteristics of functional IVD may lead to the development of novel and efficacious therapeutics for IVDD management. While human primary NP cells (NPCs) and AF cells (NPCs) are theoretically applicable and optimal for IVD tissue engineering,^[^ ^19^, ^20^ ^]^ primary AFCs and NPCs are arduous to obtain, yet with limited life span, further hampering their potential use in preclinical and clinical studies. Thus, the major challenges in effective IVD regeneration are to procure healthy NPCs and AFCs for extensive cellular phenotype studies and for the identification of the most appropriate biological factors that maintain IVD health by supporting cell survival and promoting the production of highly hydrated extracellular matrix of the IVD structure.

Here, we successfully established an IVD regeneration model by utilizing reversibly immortalized human NPCs (iHNPCs) and AFCs (iHAFCs) that were stimulated with chondrogenic and osteogenic factors and ladened into a 3D disc‐like structure of POC/GelMA scaffolds. Specifically, human NPCs and AFCs were immortalized with hTERT, yielding iHNPCs and iHAFCs, and shown to express NPC‐specific and AFC‐specific markers, respectively. The proliferative activities of the iHNPCs and iHAFCs were effectively reversed by the FLP recombinase, and both lines were non‐tumorigenic. The iHAFCs exhibited significant osteogenic differentiation upon BMP9 stimulation, whereas the iHNPCs showed chondrogenic differentiation upon BMP2 stimulation. To explore the potential application of these cells in IVD regenerative tissue engineering, we developed an IVD regeneration model using a 3D‐printed cylindrical POC scaffold ladened with BMP9‐stimulated iHAFCs/PPCNg mix in the periphery and BMP2‐stimulated iHNPCs/GelMA mix in the center and implanted the assembled IVD regeneration construct subcutaneously in athymic nude mice for six weeks. Histologic evaluation revealed significant bone nodule formation in the peripheral (iHAFCs) region and cartilage formation in the central (iHNPCs) area, mimicking natural IVD structure. Furthermore, we developed an ex vivo intervertebral fusion model by replacing the native IVD with BMP9‐stimulated iHAFCs in rat spine segments and implanting the engineered spine fusion constructs subcutaneously in athymic nude mice. MicroCT imaging and histologic analyses revealed robust bone formation in the iHAFC‐injected segments. Collectively, our findings demonstrate that iHAFCs and iHNPCs should be used as valuable sources for studying cellular functions of IVD and developing effective approaches to IVD tissue engineering.

## 2 Results

### 2.1 Human Annulus Fibrosus Cells (HAFCs) and Nucleus Pulposus Cells (HNPCs) can be Reversibly Immortalized through the Stable Expression of hTERT

**Figure 1:**
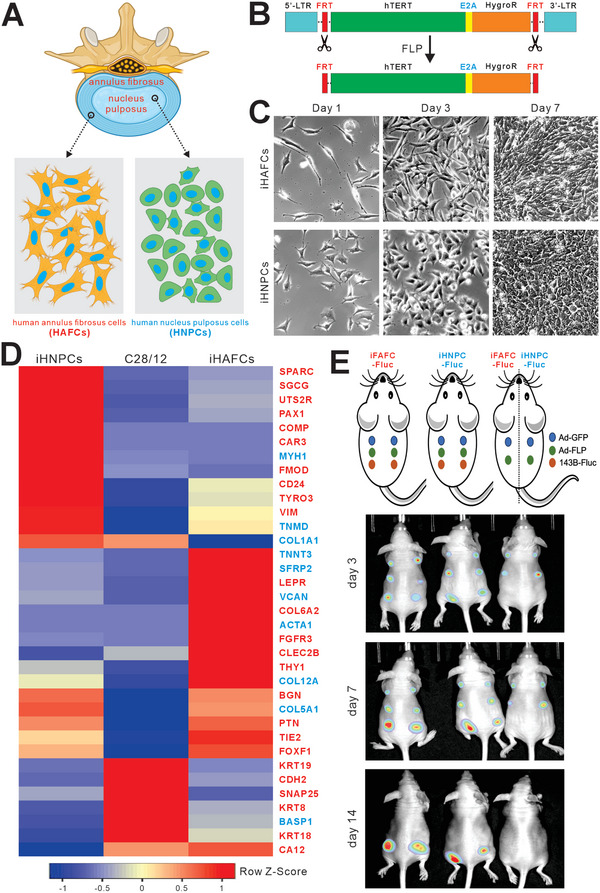
Establishment and characterization of the reversibly immortalized human nucleus pulposus cells (iHNPCs) and annulus fibrosus cells (iHAFCs). A) Schematic representation of the nucleus pulposus and annulus fibrosus regions of the human intervertebral disc. Nucleus pulposus cells (HNPCs) and annulus fibrosus cells (HAFCs) were isolated from their respective regions. B) Schematic representation of the homemade pMLV‐hTERT lentiviral vector used for immortalization. The immortalization vector expresses hTERT and self‐cleaving E2A‐linked hygromycin resistance genes, flanked with the flippase recognition target (FRT) sites for removing the hTERT and reversing immortalization using the recombinase flippase (FLP). C) Macrographic images of the immortalized cells. Primary human disc cells were immortalized with packaged hTERT lentivirus and selected for hygromycin resistance. The resulting cell lines are designated as iHAFCs and iHNPCs, respectively, both of which exhibit long‐term proliferative activity. Representative proliferation images of iHAFCs and iHNPCs at days 1, 3, and 7 after replating are shown. D) Expression of nucleus pulposus cell (NPC) and annulus fibrosus cell (AFC)‐specific genes. TqPCR analysis of AFC‐specific (in blue letters) and NPC (in red letters)‐specific gene expression compared with the human articular chondrocyte C28/12 line based on previously published studies. E) iHAFCs and iHNPCs are non‐tumorigenic. The cells stably tagged with firefly luciferase (Fluc) were infected with Ad‐GFP or Ad‐FLP and subcutaneously injected into athymic nude mice, followed by whole‐body imaging at the indicated time points. Human osteosarcoma line 143B‐Fluc was used as a control. Representative images are shown.

Primary HAFC and HNPC cells were isolated from human intervertebral disc annulus fibrosus and nucleus pulposus (**Figure** **1**A). Since the primary cells had limited life span, we sought to immortalize them using our homemade lentiviral vector pMLV‐hTERT that expresses hTERT and self‐cleaving E2A linked hygromycin resistance genes, flanked with the flippase recognition target (FRT) sites for removing the hTERT and reversing immortalization using the recombinase flippase (FLP) (Figure 1B). After selection with hygromycin B (0.3 mg mL^−1^, Invitrogen) for 7–10 days, the resultant immortalized AF and NP cells were designated as iHAFCs and iHNPCs, respectively. Both cell lines were passaged for >30 generations. Morphologically, iHAFCs were fibroblast‐like grew faster than iHNPCs, and were more round in shape (Figure 1C).

### 2.2 iHAFCs and iHNPCs Express Distinct Sets of Marker Genes and are Non‐tumorigenic

It has been well established that the AF and NP cells express distinct marker genes although it is generally speculated that AF cells are osteoblast‐like while NP cells are more chondrogenic.^[^ ^21^, ^22^, ^23^, ^24^ ^]^ Using an established human articular chondrocyte line C28/12, we conducted a comprehensive qPCR analysis of the reported AF and NP‐specific marker genes that were selected from the reported studies (Figure 1D). Compared to C28/12 cells, 7 of the 10 AF‐specific genes were significantly higher in iHAFCs, and 16 of the 25 NP‐specific genes were significantly higher in iHNPCs. When iHAFCs and iHNPCs were compared, 5 of the 10 AF‐specific genes were significantly higher in iHAFCs, and 11 of the 25 NP‐specific genes were significantly higher in iHNPCs. These results demonstrated that the established iHAFCs and iHNPCs represent distinct cell types and express the AF‐specific and the NP‐specific marker genes, respectively.

We also tested the tumorigenic potential of the immortalized cells with or without removing hTERT. To track the in vivo cell proliferation status, we used a retroviral vector and stably tagged iHAFCs and iHNPCs with firefly luciferase (Fluc), generating iHAFC‐Fluc and iHNPC‐Fluc lines. Human osteosarcoma line 143B‐Fluc stabling expressing Fluc was used as a positive control. Subconfluent Fluc‐labelled cells were infected with Ad‐FLP or Ad‐GFP for 16h, and collected for subcutaneous injection into the flanks of athymic nude mice. Whole‐body optical imaging of the injected animals was performed on days 3, 7, and 14. The bioluminescent imaging results indicated that the signals for the iHAFC‐Fluc and iHNPC‐Fluc groups were weaker at day 7, compared with the 143B‐Fluc group, and the masses and signals disappeared in the iHAFC‐Fluc and iHNPC‐Fluc groups at day 14, while the 143B‐Fluc group grew significant masses and yielded strong signals (Figure 1E). We monitored the animals for up to 4 weeks and did not find any detectable masses in both iHAFC‐Fluc and iHNPC‐Fluc groups either infected with Ad‐FLP or Ad‐GFP. Collectively, these results demonstrate that the hTERT‐immortalized iHAFCs and iHNPCs are non‐tumorigenic.

We further tested the reactive oxygen species (ROS) levels following adenoviral infection in vitro. Early‐stage adenoviral infections induced a transient ROS response in the cells; however, this effect largely subsided within 72 h post‐infection (Figure S1, Supporting Information). Additionally, to assess in vivo inflammation and oxidative stress reaction, we performed immunohistochemical (IHC) staining on subcutaneously injected samples. Markers included CD68 for inflammation and NF‐κB for oxidative stress. The results indicated that apart from macrophage aggregation due to foreign body reactions, no significant positive staining for CD68 or NF‐κB was observed in the iHAFC osteogenesis or iHNPC chondrogenesis samples (Figure S2, Supporting Information).

### 2.3 FLP‐mediated Removal of hTERT Effectively Limits the Proliferative Potential of iHAFCs and iHNPCs

**Figure 2:**
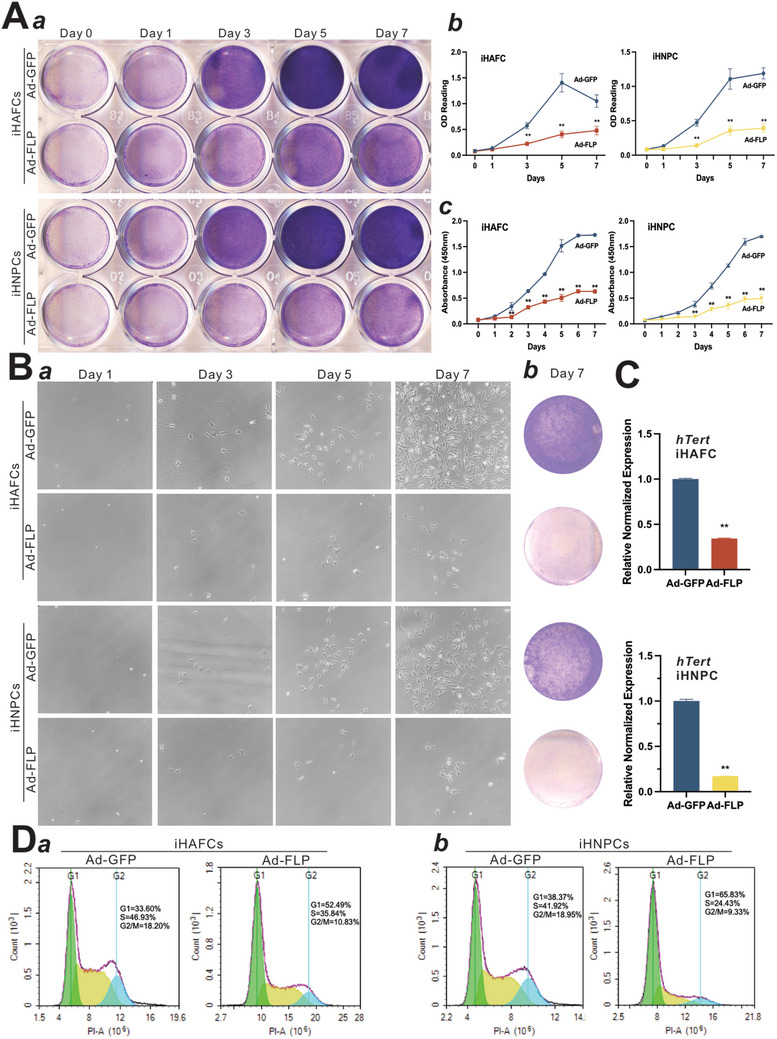
The immortalization of iHAFCs and iHNPCs is reversible. A) Cell viability and proliferation upon the removal of hTERT in iHAFC/iHNPC. Subconfluent iHAFC and iHNPC cells were infected with Ad‐FLP or Ad‐GFP. Crystal violet staining a) and quantitative OD reading analysis b) were performed at days 0, 1, 2, 3, 5, and 7. c) CCK‐8 quantitative assay was performed at days 0 to 7. Representative images are shown. “**” *p <* 0.01 compared with that of the Ad‐GFP group. B) Colony formation analysis after the removal of hTERT. Subconfluent iHAFC and iHNPC cells were infected with Ad‐FLP or Ad‐GFP for 72 h and replated into 12‐well plates at a low density (≈50 cells per well). Macrographic images a) were taken on days 1, 3, 5, and 7 after replating, and the crystal violet staining b) was performed on day 7. Representative images are shown. C) hTERT expression upon Ad‐FLP infection. Subconfluent iHAFC and iHNPC cells were infected with Ad‐FLP or Ad‐GFP for 48 h, and the total RNA was isolated from the infected cells for TqPCR analysis using hTERT‐specific primers. “**” *p <* 0.01, compared with that of the Ad‐GFP group. D) Cell proliferation assessed by cell cycle analysis. Subconfluent iHAFC and iHNPC cells were infected with Ad‐FLP or Ad‐GFP for 72 h and then collected for FACS analysis. Assays were done in triplicate, and representative results are shown. “**” *p<*0.01, compared with that of the Ad‐GFP group.

To analyze the immortalization reversibility, we infected the iHAFCs and iHNPCs with Ad‐FLP and assessed cell viability and cell proliferation upon the removal of hTERT. Specifically, subconfluent cultures of iHAFCs and iHNPCs were infected with Ad‐FLP or Ad‐GFP. Crystal violet staining and quantitative OD reading analysis were performed on days 0, 1, 2, 3, 5, and 7, indicating that FLP‐induced cells exhibited weaker viability than GFP control (**Figure** **2**A, panels ab). CCK‐8 quantitative assays were performed to further validate the results of cell proliferation (Figure 2A, panels c).

We further tested the colony proliferation capability after the removal of hTERT. Subconfluent iHAFCs and iHNPCs were infected with Ad‐FLP or Ad‐GFP for 72 h. The infected cells were replated into 12‐well plates at a low density (≈50 cells per well). Morphologic images were taken on days 1, 3, 5, and 7 after replating (Figure 2B, panel a), and crystal violet staining was performed on day 7 (Figure 2B, panel b). These results indicated that FLP‐induced cells exhibited a significantly slower proliferation rate than GFP control.

To verify the efficiency of FLP‐mediated hTERT removal, we performed TqPCR of hTERT expression in the iHAFCs and iHNPCs infected with Ad‐FLP or Ad‐GFP (as the control). After Ad‐FLP infection, the hTERT expression was significantly decreased, compared with that of GFP control (Figure 2C). Furthermore, cell cycle analysis (Figure 2D) revealed that compared with GFP control, Ad‐FLP infected cells exhibited significantly increased G1 phase (33.60% to 52.49% in iHAFCs, 38.37% to 65.83% in iHNPCs), decreased S phase (46.93% to 35.84% in iHAFC, 41.92% to 24.43% in iHNPC), and decreased G2 phase (18.20% to 10.83% in iHAFCs, 18.95% to 9.33% in iHNPCs). Collectively, these results strongly indicate that the iHAFCs and iHNPCs possess long‐term proliferative capability that can be effectively reversed by FLP recombinase‐mediated removal of hTERT.

### 2.4 iHAFCs Exhibit High Osteogenic Potential upon BMP9 Stimulation

**Figure 3:**
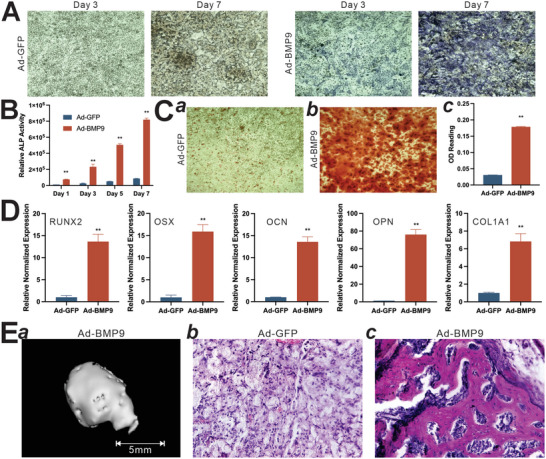
iHAFCs exhibit high osteogenic potential upon BMP9 stimulation. A,B) Early osteogenic marker alkaline phosphatase (ALP) activity induced by BMP9 in iHAFCs. Subconfluent iHAFCs were transduced with Ad‐GFP or Ad‐BMP9. Qualitative histochemical analysis or quantitative measurement of ALP activity was determined at days 1, 3, 5, and 7. “**” *p<*0.01, compared with that of the Ad‐GFP group. C) BMP9‐induced mineralized matrix formation in iHAFCs. Subconfluent iHAFCs were transduced with Ad‐GFP or Ad‐BMP9, and cultured in a mineralization medium for 14 days, followed by alizarin red S staining (a and b). Representative results are shown. The stained mineral nodules were dissolved and quantified using OD reading (c). “**” *p<*0.01, compared with that of the Ad‐GFP group. D) BMP9‐induced expression of osteogenic regulators and markers in iHAFCs. Subconfluent iHAFCs were infected with Ad‐GFP or Ad‐BMP9 and collected at 48 h after infection. Total RNA was isolated from the infected cells and subjected to TqPCR analysis of osteogenic regulators RUNX2 and OSX, and late osteogenic markers of OCN, OPN, and COL1A1. “**” *p<*0.01, compared with that of the Ad‐GFP group. E) Ectopic bone formation of BMP9‐stimulated iHAFCs. Subconfluent iHAFCs were infected with Ad‐GFP or Ad‐BMP9 and collected 36 h after infection. Cells were resuspended with thermosensitive polymer PPCNg and injected subcutaneously into the flanks of athymic nude mice. a) Masses were harvested 4 weeks after injection, followed by fixation and micro‐CT imaging. No ossified masses were detected by microCT imaging in the Ad‐GFP group. The samples were decalcified, embedded, sectioned, and subjected to b) H&E staining of the GFP group and c) BMP9 group. Representative images are shown.

We previously demonstrated that BMP9 is the most osteogenic factor among the 14 types of BMPs.^[^ ^25^, ^26^, ^27^ ^]^ Here, we found that upon transduction with Ad‐BMP9, iHAFCs underwent effective osteogenic differentiation. The early osteogenic marker alkaline phosphatase (ALP) activities were induced as early as day 1 and significantly increased on day 7, compared with that of the Ad‐GFP group (**Figure** **3**A). The quantitative ALP assays further confirmed these results, showing that the ALP of BMP9‐stimulated iHAFCs was 7 to 10‐fold higher than that of the GFP group on days 1, 3, 5, and 7 (Figure 3B). It is noteworthy that we conducted similar osteogenic assays in the iHNPCs and found that showed some, but limited osteogenic potential in vitro after BMP9 stimulation (Figure S3A–E, Supporting Information),

The capabilities of iHAFCs formed mineralized matrix were assessed by Alizarin Red S staining. After culture in a mineralization medium for 14 days, significant numbers of mineralized nodules were observed in the BMP9 group (Figure 3C, panel a versus b). Quantitative analysis indicated that BMP9 induced at least a 6‐fold increase of mineralized matrix compared to that of GFP control (Figure 3C, panel c). We further analyzed the expression of osteogenic regulators and markers. After BMP9 stimulation, the expression of RUNX2, OSX, OCN, OPN, and COL1A1 were significantly up‐regulated, compared with that of GFP control (Figure 3D).

We further assessed whether the iHAFCs could form new bone in vivo. Ad‐GFP or Ad‐BMP9 transduced iHAFCs were resuspended with thermosensitive polymer PPCNg and injected into athymic nude mice subcutaneously. The masses retrieved from the Ad‐BMP9 group, but not the Ad‐GFP group, exhibited apparent bone formation upon micro‐CT imaging analysis (Figure 3E, panel a). Histologic analysis of the retrieved masses revealed that BMP9 induced robust trabecular bone formation, while the GFP group only showed residual PPCNg polymer and injected cells (Figure 3E, panel b vs c). It is worthy pointing out that BMP9 induced no apparent new bone formation in the iHNPCs (Figure S3F, Supporting Information). Collectively, these results demonstrate that iHAFCs exhibit high osteogenic potential upon BMP9 stimulation both in vitro and in vivo.

### 2.5 iHNPCs Exhibit High Chondrogenic Potential upon BMP2 Stimulation

**Figure 4:**
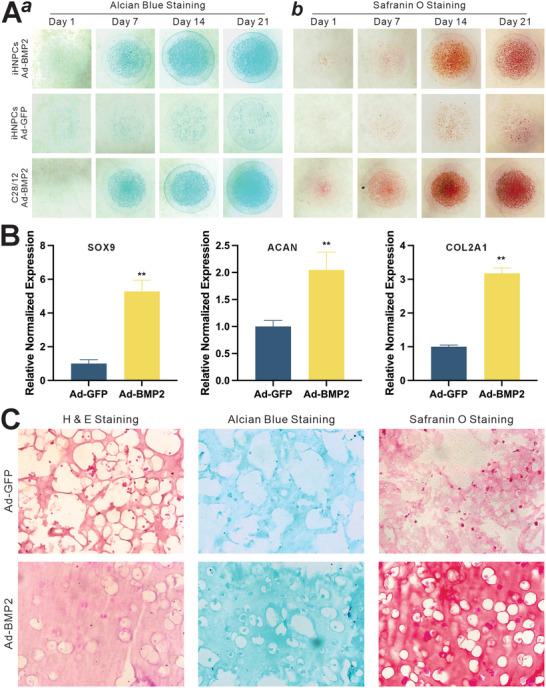
iHNPCs exhibit high chondrogenic potential in the chondrogenic differentiation medium or upon BMP2 stimulation. A) Micromass formation. Subconfluent iHNPCs and C28/12 cells were transduced with Ad‐GFP or Ad‐BMP2 for 36 h, collected, and resuspended at 5×10^6^ cells mL^−1^. 10µL drops were applied in a 48‐well plate and cultured in chondrogenic differentiation medium (CDM). Alcian blue staining a) and Safranin O staining b) were carried out at days 1, 7, 14, and 21. Representative results are shown. B) BMP2‐induced expression of chondrogenic regulators and markers in iHNPCs. Subconfluent iHNPC were infected with Ad‐GFP or Ad‐BMP2, and cultured in CDM. At 48h after infection, total RNA was isolated from the infected cells and subjected to TqPCR analysis of chondrogenic regulators/markers of SOX9, ACAN, and COL2A1. “**” *p<*0.01, compared with that of the Ad‐GFP group. C) In vitro GelMA 3D culture. Subconfluent iHNPCs were transduced with Ad‐GFP or Ad‐BMP2 for 36 h, collected, and resuspended using 10% GelMA at 5 × 10^6^ cells mL^−1^. 100 µL GelMA‐cell mix was pipetted into a sterilized circle mold and cured with UV light for 30 seconds to solidify the GelMA disc‐like 3D structure. GelMA 3D hydrogels were cultured in CDM (BMP2) and harvested at day 21, followed by fixation and section for H&E staining, Alcian blue staining, and Safranin O staining. Representative results are shown.

BMP2 is known able to induce chondrogenic differentiation in mesenchymal stem cells in vitro.^[^ ^28^, ^29^ ^]^ Micromass culture revealed that when cultured in a chondrogenic medium and stimulated with BMP2, iHNPCs were shown to undergo effective chondrogenic differentiation, compared with the C28/12 (positive control) under the same condition, as evidenced by Alcian blue and Safranin O staining (**Figure** **4**A, panel a and b). In fact, the BMP2‐induced chondrogenic differentiation of iHNPCs, similar to that of C28/12 cells, exhibited a time course‐dependence, while no significant chondrogenic differentiation was observed in the GFP‐stimulated iHNPCs group (Figure 4A, panel a and b). We also analyzed the expression of chondrogenic regulators and markers. After BMP2 stimulation, the expression levels of SOX9, ACAN, and COL2A1 were significantly upregulated, compared with that of GFP control (Figure 4B).

We next used the gelatin methacryloyl (GelMA) hydrogel to test the chondrogenic potential of iHNPCs under 3D conditions. Experimentally, subconfluent iHNPCs were infected with Ad‐BMP2 or Ad‐GFP and then harvested and mixed with GelMA hydrogel to form a 3D disc‐like structure, which was cultured in a chondrogenic medium for 21 days. The GelMA discs were harvested, fixed, and subjected to H&E staining, Alcian blue staining, and Safranin O staining. H&E staining showed apparently chondrocyte presence in the Ad‐BMP2 group, compared with that in the Ad‐GFP group (Figure 4C, left column). Both Alcian blue staining and Safranin O staining showed a stronger signal in the Ad‐BMP2 group than that in the Ad‐GFP group (Figure 4C, mid and right columns), indicating that iHNPCs can undergo chondrogenic differentiation effectively and produce a significant amount of extracellular matrix. Interestingly, when the same experiments were performed using the iHAFCs, no apparent chondrogenic differentiation was observed (Figure S3G, Supporting Information). Collectively, these results demonstrate that iHNPCs, but not iHAFCs, can undergo effective osteogenic differentiation upon BMP2 stimulation.

### 2.6 Citrate‐based Scaffold POC is Biocompatible with Both iHAFCs and iHNPCs

**Figure 5:**
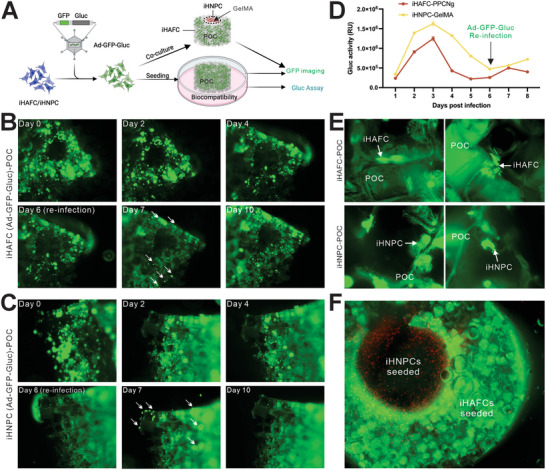
iHAFCs and iHNPCs are biocompatible with POC scaffold, and PPCNg and GelMA hydrogels in an intervertebral disc (IVD)‐like structure. A) The experimental design of a cell‐loaded IVD‐like construct. Subconfluent iHAFCs and iHNPCs were infected with Ad‐GFP‐Gluc which co‐expresses both GFP and GLuc. The cells were harvested and loaded onto the POC scaffold with GelMA (iHNPCs) or PPCNg (iHAFCs) submerged in a culture medium. The cell‐laden scaffolds were reinfected with Ad‐GFP‐GLuc on day 6. GFP signal levels B,C) and GLuc activity (relative unit, RU) D) were assessed at the indicated time points. Representative images are shown. E) POC scaffold is biocompatible and provides a 3D culture environment. White arrows indicate newly infected cells at a high magnification. F) Assembly of an IVD‐like structure by co‐seeding GFP‐labeled iHAFCs (periphery, PPCNg, green) and RFP‐labeled iHNPCs (center, GelMA, red) using the POC scaffold. Representative images are shown.

Since cell‐friendly scaffold materials are essential for effective cell‐based tissue engineering, we tested the biocompatibility of the POC scaffold with the iHAFCs and iHNPCs. Experimentally, we took advantage of the convenient qualitative (GFP signal) and quantitative (secreted Gluc activity) features of the Ad‐GFP‐Gluc vector and infected iHAFCs and iHNPCs mixed with PPCNg and seeded on to the GelMA 3D structures submerged in culture medium (**Figure** **5**A). GFP signal level and Gluc activity served as surrogates of cell survival. The cell‐laden scaffolds were reinfected with Ad‐GFP‐GLuc on day 6. We found both iHAFCs and iHNPCs tolerated the POC scaffolds and could survive for more than 10 days. Upon reinfection with Ad‐GFP‐Gluc at day 6, reemergent GFP signals were observed overnight (Figure 5B,C), and Gaussia luciferase activity was partially restored (Figure 5D), suggesting the seeded cells were still alive. High magnifications showed that the seeded cells attached to the POC scaffold in different structures, shapes, and dimensions (Figure 5E). Co‐seeding experiments indicated that cells attached well with iHNPC‐GelMA in the central part and iHAFC‐PPCNg in the peripheral part of the POC disc‐like 3D structures (Figure 5F). Collectively, these results demonstrate that the POC scaffold materials were cell‐friendly and suitable for cell‐based tissue engineering studies.

### 2.7 An Effective Intervertebral Disc (IVD) Regeneration Model can be Established by Co‐seeding BMP2‐stimulated iHNPCs and BMP9‐stimulated iHAFCs on the POC Scaffold

**Figure 6:**
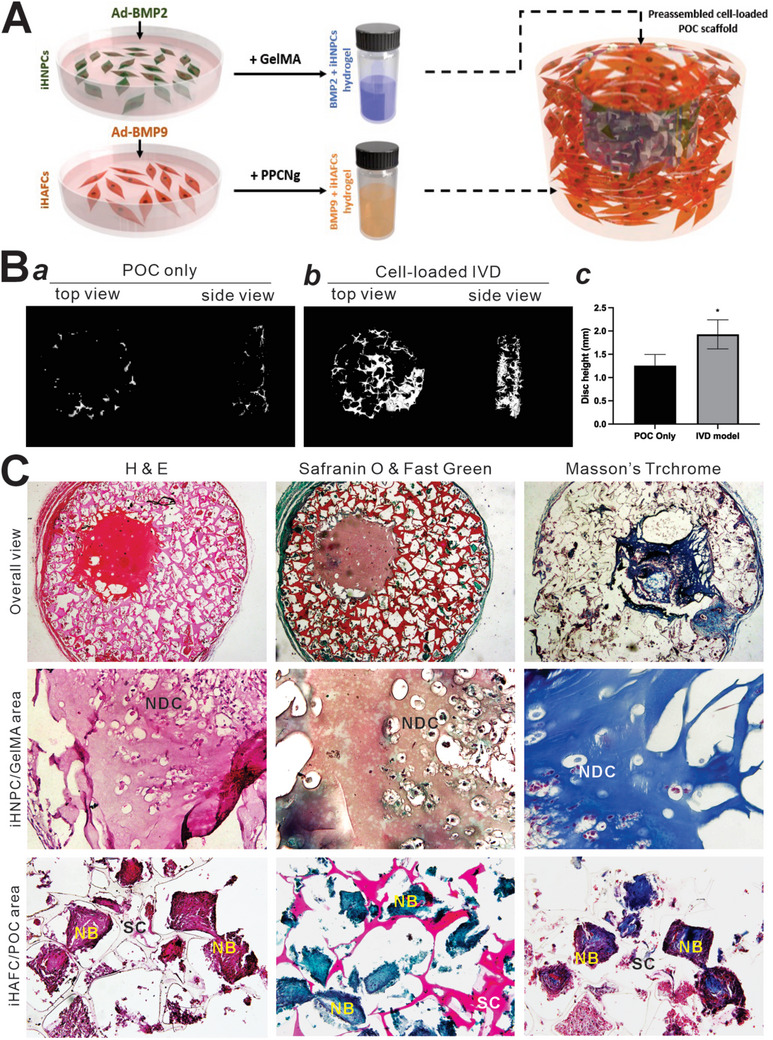
The iHAFCs and iHNPCs are desirable cell sources for intervertebral disc (IVD) tissue engineering. A) The experimental design of biologics‐treated cell‐loaded IVD tissue engineering model. In this model, iHAFCs are infected with Ad‐BMP9 while iHNPCs are with Ad‐BMP2. The POC scaffold is a 3D‐printed cylindric construct with a central cavity. BMP2‐stimulated iHNPCs/GalMA hydrogel will be loaded to the central cavity and cured using UV light, while BMP9‐stimulated iHAFCs/PPCNg hydrogel mix is loaded to the periphery, top and bottom of the POC disc, followed by subcutaneous implantation into the flanks of athymic nude mice. POC and hydrogels only (without cells) and the cells infected with Ad‐GFP are used as negative controls. B) As proof‐of‐principle experiments, multiple IVD‐like constructs were assembled according to this design and implanted into athymic nude mice subcutaneously. Animals were sacrificed at 6 weeks after implantation. a,b) Implanted masses were retrieved for micro‐CT imaging. Representative results are shown. c) The heights of the retrieved POC discs were quantitatively calculated based on 3D reconstruction from micro‐CT imaging data. “*” *p<*0.05. C) The retrieved masses were decalcified and subjected to H&E staining, Safranin O/fast green staining, and Masson's trichrome staining. Representative results are shown. NB, new bone; SC, scaffold; NDC, NPC‐derived cartilage/chondrocytes.

To mimic the structural and cellular components of IVD, we established an IVD tissue engineering model the 3D‐printed POC scaffold ladened with iHAFCs and iHNPCs. Experimentally, iHAFCs were infected with Ad‐BMP9 and iHNPCs with Ad‐BMP2. POC scaffold was 3D‐printed as a cylindrical disc‐like structure in a central void space. BMP2‐stimulated iHNPCs were loaded in the central cavity with GelMA hydrogel and cured using UV light, while BMP9‐stimulated iHAFCs were loaded in the periphery, top, and bottom with PPCNg immediately prior to subcutaneous implantation into the flanks of athymic nude mice (**Figure** **6**A). Ad‐GFP‐infected iHAFCs and iHNPCs, or POC only were used as negative controls. The animals were sacrificed at 6 weeks after implantation. The implanted masses were retrieved for micro‐CT imaging, and the 3D reconstructions of the implanted IVD model exhibited significant bone formation in the BMP9/iHAFCs and BMP2/iHNPCs co‐seeding group (IVD model group), compared with the GFP‐treated cell group or POC only group (Figure 6B, panel a and b). At the same threshold, the IVD model yielded much higher total bone volume in the periphery area than that of POC scaffold. Furthermore, the average height of the IVD model was significantly bigger than that of POC only group (Figure 6B, panel c), suggesting that that BMP9‐stimulated iHAFCs may effectively form new bone that improved the mechanical strength of the IVD model.

The retrieved IVD model samples were shown to preserve their shape well. H&E staining revealed that new bone nodules were formed in the peripheral area where the Ad‐BMP9‐infected iHAFCs were seeded, while cartilage‐like tissue was found in the central area where the Ad‐BMP2‐infected iHNPCs were loaded (Figure 6C, left column). Safranin O/fast green staining and Masson's trichrome staining further confirmed the presence of cartilage‐like tissue in the central region and mature bone in the periphery area of the IVD model (Figure 6C, mid and right columns). These results demonstrate the feasibility of establishing an IVD regeneration model using the POC scaffold seeded with chondrogenic factor‐stimulated iHNPCs and osteogenic factor‐stimulated iHAFCs.

### 2.8 iHAFCs Function as an Effective Cell‐based Therapy in an Intervertebral Fusion Model

**Figure 7:**
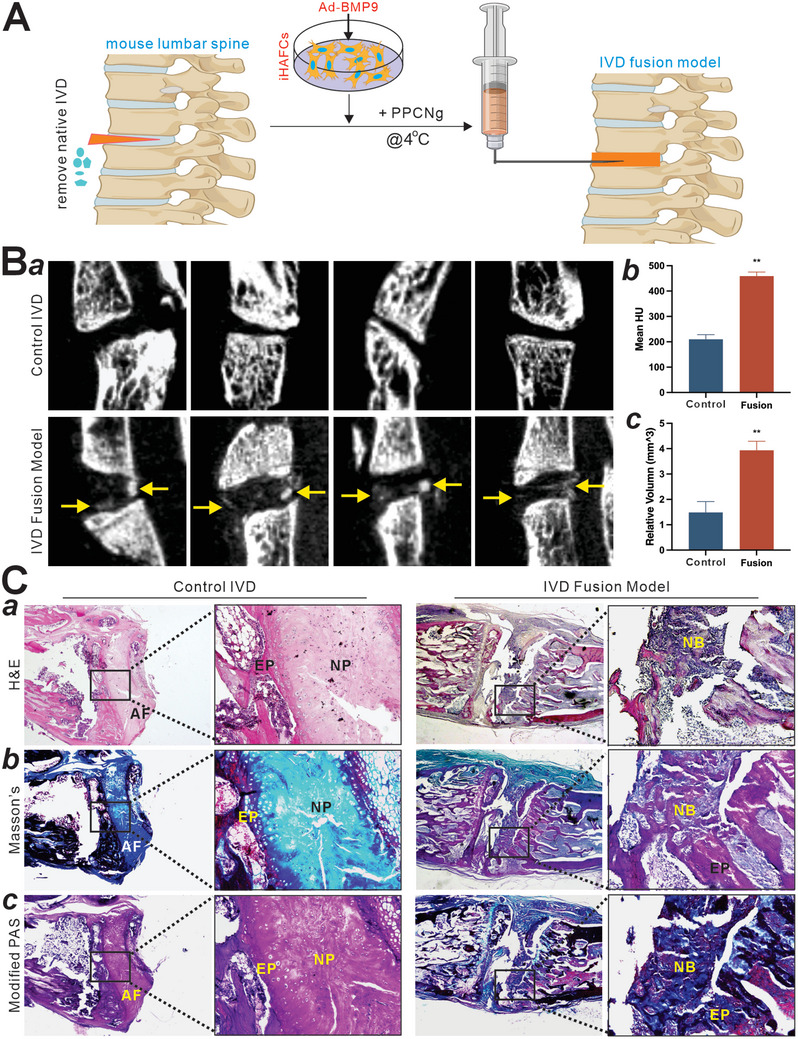
The iHAFCs provide effective intervertebral fusion. A) The experimental design of an ex vivo model of cell and gene‐based intervertebral fusion. In this model, lumbar spine segments are harvested from freshly sacrificed rats, and the targeted intervertebral discs (IVD) are removed. Meanwhile, iHAFCs are infected with Ad‐BMP9 or Ad‐GFP for 36 h, collected and resuspended with thermosensitive polymer PPCNg, followed by carefully injected into the pre‐emptied IVD void space, immediately prior to subcutaneous implantation into the flanks of athymic nude mice. Segments without IVD removal or injected with Ad‐GFP‐infected iHAFCs serve as negative controls. B) Using this model, multiple ex vivo IVD fusion constructs were assembled and implanted subcutaneously into athymic nude mice. Animals were sacrificed at 6 weeks after implantation. Implanted spine segments were a) retrieved for micro‐CT imaging, b) followed by quantitative analyses of mean new bone density (HU), and c) relative volume of new bone in the projected IVD region. Newly formed bone is indicated by arrows. Representative results are shown. “**” *p<*0.01, compared with that of the control groups. C) Histologic and specialty staining analyses. The retrieved spine segments were fixed, decalcified, and subjected to a) H&E staining, b) Masson's trichrome staining, and c) modified PAS staining. Boxed regions are further magnified (right panel). Representative results are shown. NB, new bone; AF, annulus fibrosus; NP, nucleus pulposus; EP, endplate.

Since iHAFCs exhibited high osteogenic potential upon BMP9 stimulation, we further investigated the potential application of iHAFCs in spinal fusion. Experimentally, lumbar spine segments were dissected out from freshly sacrificed rats, and the intervertebral discs were removed and implanted into the flanks of athymic nude mice. The Ad‐BMP9 or Ad‐GFP infected iHAFCs were collected, resuspended with thermosensitive polymer PPCNg, and injected into the void space of the lumbar spine segments. Segments with intact IVD or IVD‐void without loading of any cells served as negative controls, respectively (**Figure** **7**). the animals were sacrificed at 6 weeks after implantation. Implanted segments were retrieved for micro‐CT imaging analysis that showed the presence of a new bony structure between the endplates, compared to the native IVD segments (Figure 7B, panel a). The quantitative analysis of the “IVD area” indicated that the mean bone density (Hounsfield unit, HU) and relative bone volume of the fusion group was 2 to 3‐fold higher than that of native IVD (Figure 7B panels b and c).

The retrieved samples were subjected to histologic evaluation. H&E staining showed that mature bone formation and osseous junction within the endplates were readily detected in the IVD model, while the control IVD maintained normal histologic features of annulus fibrosus and nucleus pulposus between the endplates (Figure 7C, panel a). Masson's trichrome staining revealed that mature bone was formed in the IVD fusion model (Figure 7C, panel b). Conversely, a significant decrease in cartilage tissue was found in the IVD fusion model, compared with that in the control IVD model, as evidenced by the modified PAS staining (Figure 7C, panel c; Figure S4A,B, Supporting Information). Collectively, these results demonstrated that iHAFC can serve as a desirable cell source for cell‐based ex vivo therapy for intervertebral spinal fusion.

## 3 Discussion

While IVDD is considered as a part of the normal aging process, IVDD‐associated LBP is a common health concern and poses a significant socioeconomic challenge globally. Currently, most clinical managements of IVDD and LBP focus on symptomatic care and/or surgical interventions if serious complications develop. IVD regeneration through tissue engineering may represent an innovative strategy for the clinical management of IVDD and LBP. Nonetheless, any significant improvement or breakthroughs in IVDD treatment hinges on our fundamental understanding of IVD cell biology and the underlying mechanisms through which their functions are regulated.^[^ ^8^ ^]^

Even though IVD is anatomically and histologically divided into the NP, AF, and CEP tissue types, IVD cells are rather heterogeneous even within the same tissue type as revealed by single‐cell RNA sequencing studies.^[^ ^11^ ^]^ In a normal IVD, NP has a low cell density but is rich in extracellular matrix (ECM) as collagen II represents ≈20% of the NP dry weight, while proteoglycans (PG), especially aggrecan, make up ≈50% of the NP dry weight.^[^ ^11^ ^]^ The water‐retaining aggrecan is critical for maintaining NP functions as it provides osmotic swelling pressure and maintains disc height and turgor amidst heavy compressive loads or impacts. Conversely, AF has approximately three times higher cell density and provides mechanic support by forming a strong, fibrous collagen framework of the disc to secure NP cells and proteoglycans in the ECM in place while connecting the disc to the vertebral bodies.^[^ ^11^ ^]^

While IVDD is attributed to a complex interplay between environmental, genetic factors, and mechanical damage, the cell survival capability and the health status of NPCs and their putative progenitors play an essential role in determining the onset and progression of IVD degeneration.^[^ ^30^ ^]^ During embryogenesis, NP cells originate from notochord (NC) through complex and tightly regulated transcription and signaling factors such as Shh, Sox5, Sox6, Sox9, Pax1, Pax9, Pax1, Nkx3.2, and Tbx1 to name a few,^[^ ^11^ ^]^ while notochord formation itself is tightly regulated by WNT, BMP, and Activin/Nodel signaling pathways.^[^ ^31^, ^32^, ^33^ ^]^ Interestingly, while making up the NP of early IVDs, the NC cells are reduced to a minimum population with different ratios compared to other IVD cells, and the loss of NC cells is correlated with the onset of IVD although in humans human NP, NC cells disappear in early childhood (usually 4–10 years of age). It has been speculated that NC cells may transdifferentiate into chondrocyte‐like cells in the NP, and is supported by a recent single‐cell RNA‐seq analysis of the healthy human IVDs,^[^ ^11^ ^]^ in which three chondrocyte subclusters were identified, with many cells expressing Noggin (NOG), a small group of cells expressing NC markers TBXT and KRT8 and a group of multipotent NP progenitor cells expressing PROCR and PDGFRA associated with cell signaling/stemness and mesenchymal stem cells (MSC).^[^ ^34^ ^]^ Consistent with these reports, we demonstrate that the iHNPCs expressed chondrocyte makers in addition to NPC‐specific markers. Our in vitro and in vivo experiments further revealed that iHNPCs exhibited high chondrogenic potential.

Since one of the earliest changes in IVDD is a loss of PG content and composition in the NP, resulting in reduced hydration, height, and flexibility of the disc, it is critical to maintain the viability of the NPCs and to understand the molecular mechanisms through which NPCs are regulated to produce PGs and other important ECM components. In a recent transcriptomic study, the PDGF and TGF‐β cascades were identified as important cues in the NP microenvironment.^[^ ^34^ ^]^ While not specifically targeting NPCs, numerous factors, such as PDGF‐BB, BMP7, BMP2, GDF5, IGF1, TGF‐β, and bFGF, were investigated for their effectiveness in treating IVDD in preclinical studies.^[^ ^11^ ^]^ In our study, we demonstrated that BMP2 induced robust chondrogenic differentiation of the iHNPCs. It is conceivable that our established iHNPCs can be used as a valuable cell model to identify and/or validate candidate genes and/or small molecules that induce the profound synthesis and production of NP‐specific ECM and proteoglycans.

Unlike NPCs, the AFCs play a more straightforward mechanical support role in IVDs. Nonetheless, a single‐cell RNA‐seq analysis revealed multiple clusters within bovine tail AF, representing various functions, including AF progenitor cells.^[^ ^35^ ^]^ AFs form fibrocartilaginous rings composed essentially of collagen type I fibers and are divided into two compartments, the outer AF that is primarily composed of organized type I collagen fibers with high elastic and compressive properties, and the inner AF that is a less organized transitional area between the outer AF and the NP. Given the high % of collagen type I fibers presented in the outer AF, it is conceivable that AFCs may be highly osteogenic. We previously identified BMP9 (i.e., GDF2) as the most osteogenic factor among all the BMPs both in vitro and in vivo, superior to the FDA‐approved BMP2 and BMP7.^[^ ^25^, ^26^, ^27^, ^36^ ^]^ In this study, we demonstrated that BMP9 was able to induce osteogenic differentiation and robust bone formation from the iHAFCs. Interestingly, the osteogenic potential of AFCs (especially outer AFCs) may provide strong mechanical support for IVDs but on the other hand, contribute to osteophyte formation during IVD degeneration. However, from a tissue engineering prospective, osteogenic induction of AFCs should provide strong mechanical support and osteointegration of the engineered IVDs.

With a better understanding of the IVD cellular functions (esp. NPCs and AFCs) and the identification of factors that govern the proliferation and differentiation of NPCs and AFCs, we will be better positioned to carry out IVD regeneration through tissue engineering should become more efficacious and clinically feasible. In our proof‐of‐principle study, we investigated the utilization of citrate‐based biodegradable, biocompatible scaffold materials POC (FDA‐approved) and PPCN, along with the widely used gelatin‐based hydrogel GelMA and demonstrated their favorable utility in our preclinical IVD regeneration model. It is conceivable that many other types of scaffolds can be used for IVD regeneration. Furthermore, the knowledge obtained from studying IVD cells may lead to the development of other innovative strategies for potential clinical management of IVDD, such as cell‐based therapy, factor‐based therapy, gene therapy, and extracellular vesicle therapies.^[^ ^11^, ^30^ ^]^

Lastly, it is noteworthy that we established the hTERT‐immortalized human NPCs and AFCs, and carried out the proof‐of‐principle studies of preclinical IVD regeneration. We demonstrated that immortalization was reversible and the immortalized iHAFCs and iHNPCs were non‐tumorigenic. More importantly, the immortalized cells expressed the cell type‐specific markers and retained intrinsic cellular functions. Thus, the iHAFCs and iHNPCs can serve as valuable surrogates of the respective primary cell types, which enables and/or facilitates basic and translational studies on IVD cell biology and therapies. We have employed similar reversible immortalization technologies to investigate a wide range of human and mouse cell types for their functions and/or preclinical applications, especially in regenerative medicine.^[^ ^31^, ^37^, ^38^ ^]^

However, several limitations must be acknowledged. First, while hTERT‐mediated immortalization ensures long‐term cell viability and stable phenotypic characteristics, it does not fully replicate the cellular and molecular changes associated with the natural aging process, such as telomere shortening, cellular senescence, and age‐related alterations in signaling pathways, which may limit their application in some studies. Second, the POC scaffold used in this study, although functional, requires further optimization to meet the mechanical and physicochemical properties necessary for clinical applications, particularly for load‐bearing environments. Additionally, while in vivo experiments demonstrated the differentiation and functional potential of the immortalized cells, the lack of comprehensive analysis on long‐term inflammation, oxidative stress, and mechanical stimulation limits the understanding of how these factors influence scaffold integration and tissue regeneration. Future studies should aim to address these limitations by incorporating age‐mimicking models, enhancing scaffold properties, and investigating long‐term in vivo outcomes under physiologically relevant conditions.

## 4 Conclusions

To evaluate the potential cell and scaffold‐based intervertebral disc (IVD) tissue engineering, we reversibly immortalized human healthy nucleus pulposus cells (iHNPC) and annulus fibrosus cells (iHAFC) by stably expressing hTERT, which can be reversed by the FLP recombinase and non‐tumorigenic. The iHAFC exhibited osteogenic differentiation upon BMP9 stimulation, while the iHNPC showed chondrogenic differentiation upon BMP2 stimulation. The IVD regeneration tissue engineering model was developed via a 3D‐printed cylindrical POC scaffold laden with BMP9‐stimulated iHAFCs/PPCNg mix in the peripheral region and BMP2‐stimulated iHNPCs/GelMA hydrogel mix in the central area. Histologic analysis revealed significant bone nodule formation in the peripheral part and chondrogenic differentiation in central areas, mimicking natural IVDs. Furthermore, we developed an ex vivo intervertebral fusion model by replacing the native IVD with BMP9‐stimulated iHAFCs in rat spine segments and implanting the engineered spine fusion constructs subcutaneously in athymic nude mice. MicroCT imaging and histologic analyses revealed robust bone formation in the iHAFC‐injected segments. Collectively, our findings demonstrate that iHAFCs and iHNPCs should be used as valuable sources for studying cellular functions of IVD and developing effective approaches to IVD tissue engineering.

## 5 Experimental Section

### Cell Culture and Chemicals

Primary human nucleus pulposus (NP) cells (or HNPCs) and annulus fibrosus (AF) cells (or HAFCs) were purchased from ScienCell Research Laboratories (Carlsbad, CA). HEK‐293 cells were obtained from the American Type Culture Collection (ATCC, Manassas, VA). 293pTP, RAPA and 293GP were derived from HEK‐293 cells as previously described.^[^ ^39^, ^40^, ^41^ ^]^ Human chondrocyte line C28/12^[^ ^42^ ^]^ was obtained from Sigma‐Aldrich USA. All cells were cultured in DMEM supplemented with 10% fetal bovine serum (FBS, Gemini Bio‐Products), 100 U mL^−1^ penicillin, and 100µg mL^−1^ streptomycin at 37 °C in 5% CO_2_ as described.^[^ ^43^, ^44^ ^]^ Unless indicated otherwise, chemicals were obtained from Thermo Fisher Scientific (Waltham, MA) or Millipore Sigma (St. Louis, MO).

### Construction and Amplification of the Recombinant Adenoviruses

All recombinant adenoviruses were constructed using either AdEasy technology or OSCA technology as previously described.^[^ ^45^, ^46^, ^47^, ^48^ ^]^ Briefly, the coding sequences of human BMP2 and BMP9, FLP recombinase from Saccharomyces cerevisiae 2 µ plasmid, and secreted Gaussia luciferase of Gaussia princeps were PCR amplified and subcloned into an adenoviral shuttle vector to generate adenoviral plasmids. Recombinant adenoviruses were packaged in 293pTP, RAPA, or 293GP cells, and amplified to high titers in HEK‐293 cells.^[^ ^48^ ^]^ Ad‐BMP2, Ad‐BMP9, and Ad‐FLP also co‐express GFP. Ad‐GFP or Ad‐RFP were used as mock control adenoviruses as described.^[^ ^49^, ^50^, ^51^ ^]^ Polybrene (final concentration at 6 µg mL^−1^) was added to enhance adenoviral transduction efficiency in the target cells as described.^[^ ^31^, ^52^ ^]^

### Establishment of the Reversibly Immortalized Human Nucleus Pulposus Cells (iHNPCs) and Annulus Fibrosus Cells (iHAFCs)

Primary human AF and NP cells (< three passages) were used to generate the immortalized cell lines. The homemade lentiviral transfer vector pMLV‐hTERT that expresses hTERT and self‐cleaving E2A linked hygromycin resistance genes, flanked with the flippase recognition target (FRT) sites, was co‐transfected with pCMV‐VSVG and psPAX2 packaging plasmids into HEK‐293 cells to produce lentiviral particles as described.^[^ ^53^, ^54^, ^55^ ^]^ Subconfluent primary human AF and NP cells were infected with the packaged hTERT lentivirus, followed by the selection with hygromycin B (0.3 mg mL^−1^, Invitrogen) for 7–10 days. The resultant immortalized human AF and NP cell pools were designated as iHAFCs and iHNPCs, respectively, which have been passaged for >30 generations. All assays in this study were conducted using cells from passages beyond the 30th generation. The immortalization phenotype can be reversed by the introduction of FLP recombinase.^[^ ^56^, ^57^ ^]^

### Touchdown‐quantitative Real‐time PCR (TqPCR)

Total RNA was isolated using NucleoZol (Takara Bio, USA) and reverse transcribed with hexamer and M‐MuLV reverse transcriptase (New England Biolabs, Ipswich, MA) as described.^[^ ^31^, ^50^, ^58^ ^]^ The cDNA products served as PCR templates. Primers for human AF and NP‐specific marker genes were designed by using the Primer3 program (Table S1, Supporting Information). TqPCR was conducted with 2x SYBR Green qPCR Master Mix (Thermo Fisher Scientific, USA) on a CFX‐Connect unit (Bio‐Rad Laboratories, Hercules, CA) as described.^[^ ^59^, ^60^, ^61^, ^62^ ^]^ All TqPCR reactions were conducted in triplicate, with ACTB used as the reference gene. Gene expression quantification was performed using the 2^−ΔΔCq^ method.

### Tumorigenic Potential In Vivo

Stable cell lines iHAFC‐Fluc and iHNPC‐Fluc that constitutively express firefly luciferase were established through retrovirus as described.^[^ ^37^, ^63^ ^]^ After Ad‐GFP or Ad‐FLP (to reverse immortalization) infection, cells were injected subcutaneously in athymic nude mice (5 × 10^6^ cells per injection site, 9 injections for each group). Human osteosarcoma line143B‐Fluc stabling expressing firefly luciferase served as the positive control. Bioluminescent imaging was performed at the indicated timepoints using the Lago X Imaging System (Spectral Instruments Imaging, Tucson, AZ) at the University of Chicago Integrated Small Animal Imaging Research Resource. Animals were anesthetized at 2% isoflurane throughout the duration of image acquisition. Imaging data were analyzed with the companion Aura Image Analysis Software.

### Cell Viability Assay

Subconfluent iHAFCs and iHNPCs were infected with Ad‐FLP or Ad‐GFP. At the indicated time points, the cells were replated into another 24‐well plate to remove dead cells. After a 4–6 h incubation, the attached cells were gently washed with PBS and stained with 0.5% crystal violet/formalin solution for 10 min. The stained cells were washed with tap water and air‐dried for scanning. For quantitative analysis, the stained cells were dissolved in 10% acetic acid and measured for absorbance at 592 nm as described.^[^ ^64^, ^65^ ^]^ Cell Counting Kit‐8 (CCK‐8) quantitative assays were performed from day 0 to 7 and absorbance at 450nm were measured.

### Alkaline Phosphatase (ALP) Activity

Subconfluent iHAFCs were transduced with Ad‐GFP or Ad‐BMP9 for 24 h and subsequently seeded in 24‐well plates. ALP activity was quantitatively assessed using the Great Escape SEAP Chemiluminescence assay kit (Takara USA Bio) and qualitatively with histochemical staining assay using a mixture of 0.1 mg mL^−1^ naphthol AS‐MX phosphate and 0.6 mg mL^−1^ Fast Blue BB salt at specified time points as described.^[^ ^66^, ^67^ ^]^ The staining images were documented under a bright field microscope.

### Matrix Mineralization Assay

Alizarin red staining was performed as described.^[^ ^68^, ^69^ ^]^ Subconfluent iHAFCs were transduced with Ad‐GFP or Ad‐BMP9 for 24 h and subsequently seeded in 24‐well plates, cultured with the mineralization medium. At the indicated time points, cells were fixed with 2.5% glutaraldehyde and stained with 0.4 % Alizarin red S for 5 min, followed by extensive washing with distilled water. The stained calcium mineral deposits were documented under a bright field microscope. The stain was dissolved in a solution of 20% methanol and 10% acetic acid in water, and quantified by OD reading at 450nm.

### Chondrogenic Micromass Formation In Vitro Culture

Subconfluent iHNPCs and C28/12 cells were infected with Ad‐GFP or Ad‐BMP2 for 36 h, collected, and resuspended into 5 × 10^6^ cells mL^−1^. 10 µL drops were applied in 48‐well plates and incubated for 2 h to attach. 0.5 mL chondrogenic differentiation medium (CDM) was added for cell culture. CDM contains DMEM supplemented with 10% ITS+ Premix Tissue Culture Supplement (Becton Dickinson), 0.1 µm dexamethasone, 1 µm ascorbate‐2‐phosphate, 10% fetal bovine serum, 100 U mL^−1^ penicillin, and 100µg mL^−1^ streptomycin. The GFP‐ iHNPCs group was considered as a negative control, the BMP2‐C28/12 cells group was considered as a positive control, and the BMP2 ‐iHNPCs group was the experimental group. On days 1, 7, 14, and 21, the micromasses were fixed with 2.5% glutaraldehyde and subjected to Alcian blue staining and Safranin O staining.

### Alcian Blue Staining and Safranin O Staining

Alcian blue staining and Safranin O and fast green staining as described.^[^ ^54^, ^61^, ^70^, ^71^, ^72^ ^]^ Briefly, cultured micromasses, and sections from in vitro GelMA 3‐D culture and the retrieved samples were fixed and stained with 1% alcian blue (pH2.5), followed by washing with 0.1 m HCl and PBS. For Safranin O staining, samples were fixed and stained with Weigert's iron hematoxylin working solution for 10 min. After being washed briefly, samples were stained with 0.05% Fast Green for 5min, and rinsed with 1% acetic acid briefly, followed by being stained in 0.1% safranin O solution for 5min and dehydrated prior to cover‐slipped. Cartilage tissue was stained orange or red.

### Gelatin Methacryloyl (GelMA) Hydrogel 3D Culture for Chondrogenic Differentiation Assay of iHNPCs

GelMA was obtained from Sigma‐Aldrich (Cat# 900741). 10% GelMA hydrogel was prepared as described.^[^ ^18^ ^]^ Subconfluent iHNPCs were transduced with Ad‐GFP or Ad‐BMP2 for 36 h, collected, and resuspended using 10% GelMA into 5 × 10^6^ cells mL^−1^. 100 µL GelMA‐cell mix was pipetted into a sterilized circle mold and cured with UV light for 30 s to form a GelMA disc‐like 3D construct. GelMA 3D constructs were cultured in a chondrogenic differentiation medium and harvested at day 21, followed by fixing and sectioning for H&E staining, Alcian blue staining, and Safranin O staining.

### Poly (1,8‐octanediol‐co‐citric acid) (POC) Scaffold Biocompatibility Assays

POC was synthesized and characterized as described.^[^ ^73^, ^74^ ^]^ The POC scaffolds were 3D‐printed as a 5 mm × 3 mm (diameter × height) disc‐like structure with a central void space of 1 mm × 2 mm (diameter × height). Subconfluent iHAFCs and iHNPCs were infected with Ad‐GFP‐Gluc for 16 h, collected, and loaded onto POC scaffolds with GelMA or PPCNg, submerged in a cell culture medium. The cell‐laden scaffolds were re‐infected with Ad‐GFP‐GLuc at day 6. GFP signal levels and GLuc activities were assessed at the indicated timepoints and served as surrogates of cell survival. Gaussia luciferase activity in the culture medium was quantified using the Secrete‐Pair Gaussia Luciferase Assay Kit (GeneCopoeia, Rockville, MD) as described.^[^ ^37^, ^49^, ^75^ ^]^

### Subcutaneous Ectopic Bone Formation of iHAFCs

The care and use of animals were approved by the Institutional Animal Care and Use Committee (IACUC) of The University of Chicago (ACUP #71328). All experimental procedures followed the approved guidelines. Athymic nude mice (Envigo, Indianapolis, IN; 6–8‐week‐old, both male and female) were used for subcutaneous injection experiments. Thermoresponsive polymer PPCNg was synthesized and characterized as described.^[^ ^16^, ^76^ ^]^ Ectopic bone formation was described previously.^[^ ^62^, ^77^ ^]^ Briefly, Ad‐BMP9, or Ad‐GFP infected iHAFCs were collected and resuspended in sterile PPCNg for subcutaneous injection into the flanks of athymic nude mice (5 × 10^6^ cells per injection, 6–8 injections per mouse). At 28 days after injection, mice were sacrificed. The subcutaneous masses at the injection sites were retrieved and subjected to microCT imaging and histologic analysis.

### Intervertebral Disc (IVD) Regeneration Model

All experimental procedures followed the approved IACUC guidelines (ACUP #71445). iHAFCs were infected with Ad‐BMP9, or Ad‐GFP, while iHNPCs with Ad‐BMP2 or Ad‐GFP. POC scaffold was 3D‐printed as a 5 mm × 3 mm disc‐like construct with a central void space (3 mm × 2 mm). The infected iHNPCs were loaded in the central cavity with 10% GelMA and cured using UV light, while the infected iHAFCs were loaded in the periphery with PPCNg immediately after subcutaneous implantation into the flanks of athymic nude mice. Animals were sacrificed at 6 weeks after implantation. The implanted masses were retrieved for micro‐CT imaging and histologic analyses.

### Intervertebral Fusion Model using the iHAFCs

Fresh lumbar spine segments were harvested from sacrificed SD rats (Envigo, Indianapolis, IN; 8‐week‐old Sprague Dawley rats, weight range 200–350 g, both male and female). Each segment contained at least three intervertebral discs (IVDs) that were removed to make the space for cell injection. iHAFCs were infected with Ad‐BMP9 or Ad‐GFP for 36 h, collected, and resuspended with thermosensitive polymer PPCNg. The IVD‐removed spine segments were subcutaneously implanted into the flanks of athymic nude mice. The PPCNg‐infected iHAFC mix was injected into the IVD‐removed cavity and cured immediately using an infrared ray heater. Segments with or without original IVD (no cell injection) served as negative controls. Animals were sacrificed at 6 weeks after implantation. Implanted spine segments were retrieved for micro‐CT imaging and histologic evaluations.

### Micro‐CT Imaging Analysis

Retrieved specimens were fixed in 10% buffered formalin and subjected to micro‐CT imaging using the X‐CUBE Preclinical CT Imager (Molecubes NV, Belgium) at The University of Chicago Integrated Small Animal Imaging Research Resource (iSAIRR) facilities. Reconstructions were performed using Amira or 3‐D Slicer software (Version 5.4.0) as described.^[^ ^78^, ^79^, ^80^ ^]^

### H&E Staining and Masson's Trichrome Staining

The retrieved specimens were fixed in 10% PBS‐buffered formalin, decalcified, and paraffin‐embedded using Leica TP1020 Automatic Tissue Processor (Leica Microsystems, Deerfield, IL). Sections were deparaffinized, rehydrated, and subjected to H&E staining as described.^[^ ^25^, ^51^ ^]^ Sections were also subjected to Masson's trichrome staining (Newcomer Supply) as described.^[^ ^26^, ^72^ ^]^

### Statistical Analysis

All experiments were performed at least three times or repeated in three batches of independent experiments. Data were analyzed using GraphPad Prism 7 and presented as the mean ± standard deviations (SD). Statistical significance was determined by one‐way ANOVA and the student's *t*‐test for comparison between groups. A value of *p <* 0.05 was considered statistically significant. Animal works were reported in line with the ARRIVE criteria.^[^ ^81^ ^]^

## Conflict of Interest

The authors declare no conflict of interest.

## Author Contributions

Y.Z. and Q.L. contributed equally to the work. L.W., T.C.H., Y.Z., Q.L. conceived and designed the study. Y.Z. and Q.L. established the immortalized cell lines. Y.J. and E.G. synthesized the 3D‐printed scaffold materials. Y.Z., C.Y., H.Z., J.Z., and Y.W. performed in vitro and in vivo experiments and collected data. Y.Z., E.G., O.M., W.Y., and G.S. performed histologic processing, figure assembly, and statistical analysis. C.L., X.W., J.L., Y.S., B.Y., Y.W., and U.Z. participated in experiments, provided essential experimental materials; and/or assisted in data analysis and interpretations. Y.Z., L.W., T.C.H., R.R.R., J.F., J.Y., L.L.S., M.J.L., and H.H.L. drafted and revised the manuscript. All authors reviewed and approved the manuscript.

## Supporting information



Supporting Information

## Data Availability

The data that support the findings of this study are available in the supplementary material of this article.
