# Supplementary material for: An Intervertebral Disc (IVD) Regeneration Model Using Human Nucleus Pulposus Cells (iHNPCs) and Annulus Fibrosus Cells (iHAFCs)
Source: Adv Healthc Mater. 2025 Mar 7;14(10):2403742. doi: 10.1002/adhm.202403742 (PMC12004445; doi:10.1002/adhm.202403742)
Supplement: Supplementary file 1 — Supporting Information [file ADHM-14-0-s001.docx]

**An intervertebral disc (IVD) regeneration model using** **human** **nucleus pulposus cells (iHNPCs) and annulus fibrosus cells (iHAFCs)**

**Running Title: Cell and gene-based IVD tissue engineering**

Yi Zhu^1,2,3#^, Qing Liu^3,4#^, Chao Yu^3,5^, Hui Zhang^3,6^, Jiamin Zhong^3,7^, Yonghui Wang^3,8^, Ou Mei^3,9^, Ethan Gerhard^10^, Wulin You^3,11^, Guowei Shen^3,12^, Changqi Luo^3,13^, Xingye Wu^3,14^, Jingjing Li^3,15^, Yi Shu^3,16^, Ya Wen^3, 17^, Usman Zeb^3,18^, Hue H. Luu^3^, Michael J. Lee^3^, Lewis L. Shi^3^, Yang Bi^16^, Jian Yang^19^, Jiaming Fan^3,7^, Russell R. Reid^3,20^, Tong-Chuan He^3,20^*, Liangyuan Wen^2^*

^1^ Department of Orthopaedic Surgery, The First Affiliated Hospital of Soochow University, Orthopaedic Institute, Soochow University, Suzhou, 215006, China

^2^ Department of Orthopaedic Surgery, Beijing Hospital, National Center of Gerontology, Institute of Geriatric Medicine, Chinese Academy of Medical Sciences & Peking Union Medical College, Beijing, 100005, China

^3^ Molecular Oncology Laboratory, Department of Orthopedic Surgery and Rehabilitation Medicine, The University of Chicago Medical Center; Chicago, IL, 60637, USA

^4^ Department of Orthopaedics, Xiangya Hospital of Central South University, National Clinical Research Center for Geriatric Disorders, Changsha, 410017, China

^5^ Department of Orthopaedic Surgery, the Affiliated University-town Hospital, Chongqing Medical University, Chongqing, 401331, China

^6^ The Breast Cancer Center, Chongqing University Cancer Hospital, Chongqing 4000430, China

^7^ Ministry of Education Key Laboratory of Diagnostic Medicine, and Department of Clinical Biochemistry, School of Laboratory Medicine, Chongqing Medical University, Chongqing 400016, China

^8^ Department of Geriatrics, Xinhua Hospital, Shanghai Jiao-Tong University School of Medicine, Shanghai 200000, China

^9^ Department of Orthopedics, Jiangxi Hospital of Traditional Chinese Medicine, Jiangxi University of Traditional Chinese Medicine, Nanchang 330006, China

^10^ Department of Biomedical Engineering, The Pennsylvania State University, University Park, PA 16802, USA

^11^ Department of Orthopaedic Surgery, Wuxi Hospital Affiliated to Nanjing University of Chinese Medicine, Wuxi 214071, China

^12^ Department of Orthopaedic Surgery, BenQ Medical Center, The Affiliated BenQ Hospital of Nanjing Medical University, Nanjing 210019, China

^13^ Department of Orthopaedic Surgery, Yibin Second People’s Hospital, Affiliated with West China School of Medicine, Yibin 644000, China

^14^ Department of Gastrointestinal Surgery, the First Affiliated Hospital of Chongqing Medical University, Chongqing 400016, China

^15^ Department of Oncology, The Affiliated Hospital of Shandong Second Medical University, Weifang 261053, China

^16^ Stem Cell Biology and Therapy Laboratory of the Pediatric Research Institute, the National Clinical Research Center for Child Health and Disorders, and Ministry of Education Key Laboratory of Child Development and Disorders, the Children’s Hospital of Chongqing Medical University, Chongqing 400016, China

^17^ School of Biomedical Engineering, Capital Medical University, Beijing 100069, China

^18^  Institute of Biotechnology and Genetic Engineering, The University of Agriculture, Peshawar 25130, Pakistan

^19^  Research Center for Industries of the Future, and Biomedical Engineering Program, School of Engineering, Westlake University, Hangzhou 310030, China

^20^  Laboratory of Craniofacial Biology and Development, Section of Plastic and Reconstructive Surgery, Department of Surgery, The University of Chicago Medical Center; Chicago, IL, 60637, USA

^#^ These authors contributed equally to the reported work

* Corresponding authors: tche@uchicago.edu; wenliangyuan1964@126.com

**Supporting Materials**

| **Table S1. List of qPCR Primers** | | | |
| --- | --- | --- | --- |
| Gene | Forward | Reverse | Accession No. |
| *ACTA1* | TCGTCCACCGCAAATGCT | ACGATGGCAGCAACGGAA | NM_001100.4 |
| *ACTB* | CTACGAGCTGCCTGACGG | GCGGATGTCCACGTCACA | NM_001101.5 |
| *BASP1* | GGGAGGCAAGCTCAGCAA | GGTCCCCTCCTCTTCCGT | NM_006317 |
| *BGN* | GACATCTCCGAGCTCCGC | TTCCGCAGTGGGCTGAAG | NM_001711.6 |
| *CA12* | GCAACCCCACTGTGCTCT | TCTCTGGGGGAAGGGTCG | NM_001218 |
| *CAR3* | GTGGCTGCTGCTGAAGGA | AGGTCGCCAGTTGCTCAC | NM_005181.4 |
| *CD24* | GCGGGCCGAGAGATAACC | AGAAAAGTCCGCGCCTCC | NM_013230 |
| *CDH2* | CTCAGGCTCCAAGCACCC | ACTGTGATGACGGCCGTG | NM_001792 |
| *CLEC2B* | GGGGGTGGGGCTTTAAGAG | TCCCCTGGCCTGTCTACC | NM_005127 |
| *COL12A1* | AGTCCAGTCGCAACCAGC | CCCCCGAGGTCCTGAAGA | NM_004370 |
| *COL1A1* | GCTCCTGGCAGCAAAGGA | CAGTGGGTCCGGGTTCAC | NM_000088 |
| *COL5A1* | GGGCCGAAATTCCCACCA | CATTCCCGGCCCGATCTC | NM_000093 |
| *COL6A2* | TGGTGCAGTACAGCCACG | GATGAGGCGGTCGTAGGC | NM_001849.4 |
| *COMP* | TGGAAGGACCCGCGAAAC | AGACCACGTTGCTGTCGG | NM_000095.3 |
| *FGFR3* | GACCTGGTGTCCTGTGCC | TCGAGGTTGTGCACGTCC | NM_000142.5 |
| *FOXF1* | GCTCGGCTTCAACCACCT | TTCATCATGCCCAGGCCG | NM_001451 |
| *GAPDH* | CAACGAATTTGGCTACAGCA | AGGGGAGATTCAGTGTGGTG | NM_002046 |
| *KRT18* | AGCCTGAGGGAGGTGGAG | GCGGTAGGTGGCGATCTC | NM_000224 |
| *KRT19* | ACTGGCAGAAACGGAGGC | AGCCGCTGGTACTCCTGA | NM_002276 |
| *KRT8* | CCAAGTGGAGCCTCCTGC | TCCTGGCCCAGAGTCTCC | NM_001256282 |
| *LERP* | GCTGGGATTACGGGCACA | GCCAATGAGGGCGGATCA | NM_002303.6 |
| *MYH1* | TGACTGTGACCTGCTGCG | GGTCCTCCACTGGGCAAC | NM_005963.4 |
| *OCN* | TGACGAGTTGGCTGACCA | AGGGTGCCTGGAGAGGAG | NM_199173 |
| *OPN* | CTGAAACCCACAGCCACA | TGTGGAATTCACGGCTGA | NM_001040058 |
| *OSX* | GCAGAGCAGGTTCCTCCA | ATGGGCTTCCCCAAAGAG | NM_152860 |
| *PAX1* | GAACTCGCGGCAGCAATG | AGCTGCCCCTAGGAGGTC | NM_006192 |
| *PTN* | AGCCCCAGAGCAGAGGAA | TCCTCTCTGGCGCTCAGT | NM_002825 |
| *RUNX2* | TGCTTCATTCGCCTCACA | CGGTGGCTGGTAGTGACC | NM_001024630 |
| *SFRP2* | CTGCGTGCAGGTGAAGGA | AGGAGGTGGTCGCTGCTA | NM_003013 |
| *SGCG* | CTGCCTCTGAGCTGCCTG | ACTTCCCATCCACGCTGC | NM_000231 |
| *SNAP25* | CCATCAGTGGCGGCTTCA | CAGGGCCATGTGACGGAG | NM_003081 |
| *SPARC* | GCAAGAAGCCCTGCCTGA | GCCGTGTTTGCAGTGGTG | NM_003118.4 |
| *TERT* | ACGTGGAAGATGAGCGTG | AACGTGGTCTCCGTGACA | NM_198253.3 |
| *THY1* | CCCGCAGACTTTCAGCCA | ATGGGGTTGGGGCATGTG | NM_006288.5 |
| *TIE2* | TGCATTGCCTCTGGGTGG | GCCTCTCCTCGAACTCGC | NM_000459.5 |
| *TNMD* | CGTCGAGGCAACCGCTAT | GGGCCACCCACCAGTTAC | NM_022144 |
| *TNNT3* | TCCAGGCCCTCATCGACA | GCCTTTTCCTCCGCCAGT | NM_006757.4 |
| *TYRO3* | CCCCCTTTACCTGCCTGC | GAGGTTTTGGGGAGCGCT | NM_006293 |
| *UTS2R* | CACGCTGCTCACCAGGAA | CTGTCAGTGGGCTGTGGG | NM_018949.3 |
| *VCAN* | CAGGTGCCTCTGCCTTCC | TGCGTCGATGGGCAAAGT | NM_004385 |

**
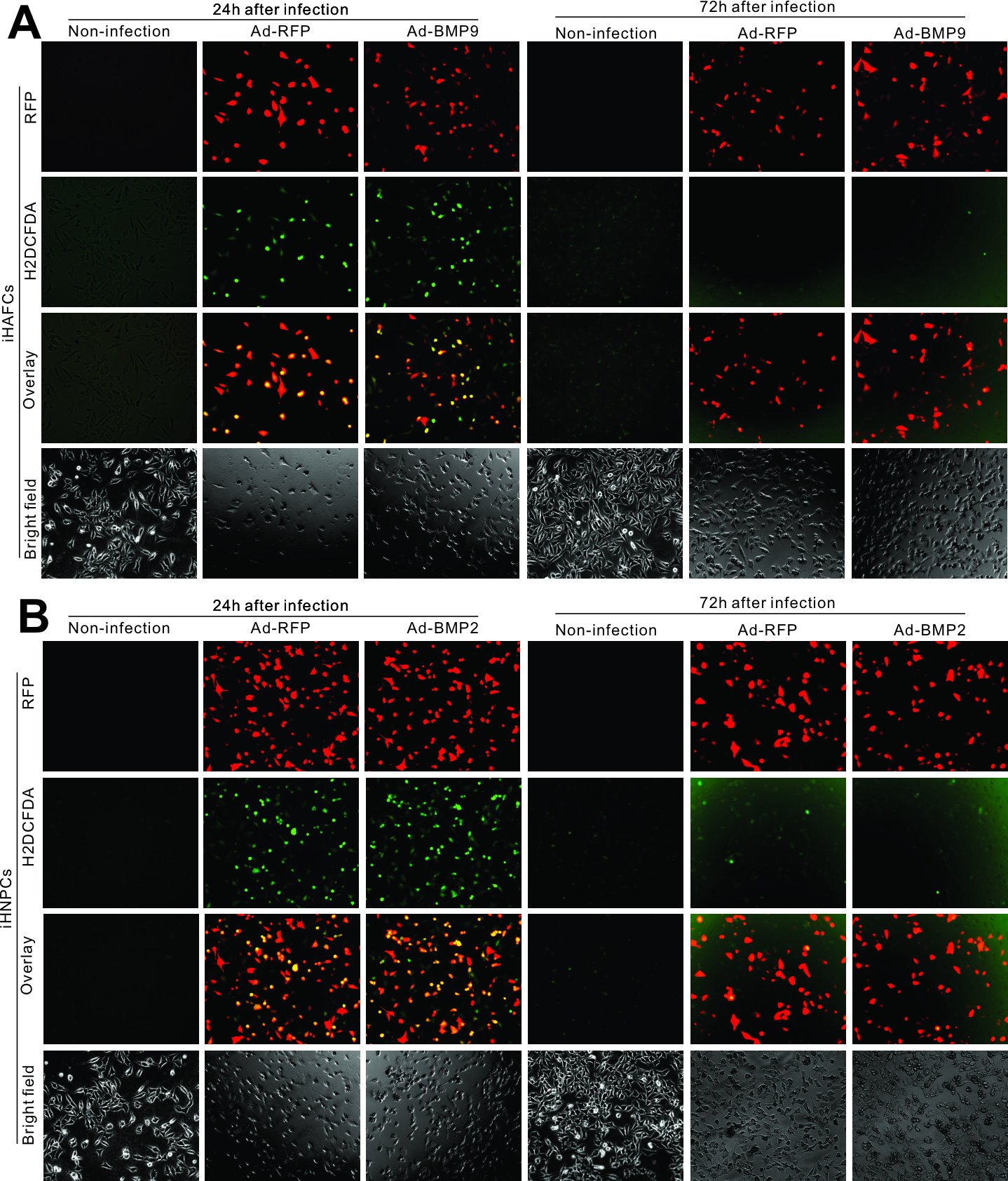
**

**Figure S1. Reactive oxygen species (ROS) levels following adenoviral infection.** iHAFCs **(A)** and iHNPCs **(B)** were grouped as non-infection, Ad-RFP infection, and Ad-BMP2/9 infection. RFP signals represented the infection status, and H2DCFDA was used to measure cellular ROS levels. Higher green fluorescence indicates a stronger reaction. The results indicated that early-stage adenoviral infections induced a transient ROS response in the cells; however, this effect largely subsided within 72 hours post-infection.

**
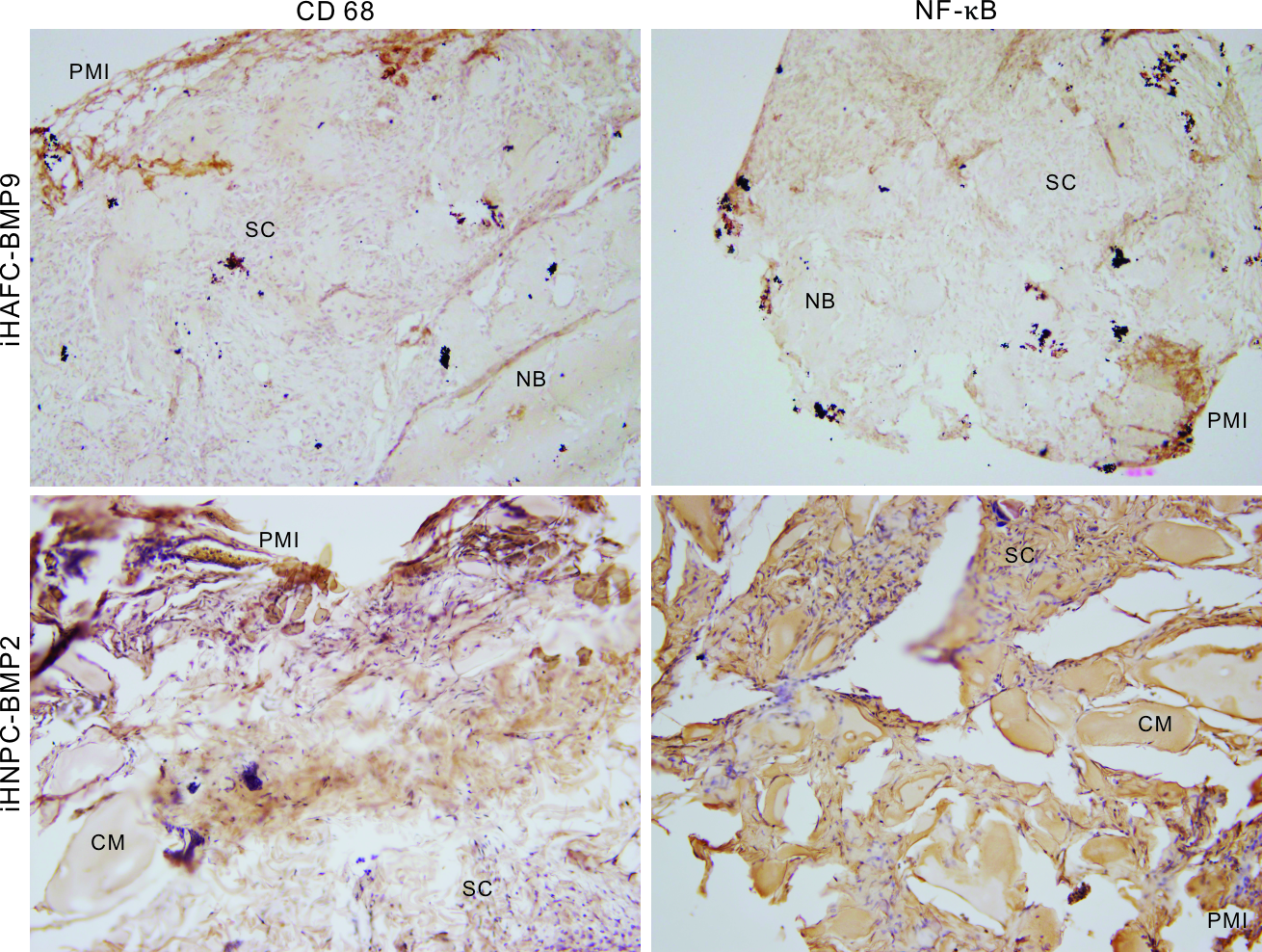
**

**Figure S2. Inflammation and oxidative stress of subcutaneous injection masses.** iHAFCs and iHNPCs were infected with Ad-BMP9 and Ad-BMP2 *in vitro*, respectively, and subcutaneously injected into nude mice 48 hours after infection. Masses were collected at week 6 for immunohistochemical (IHC) staining. Markers included CD68 for inflammation and NF-κB for oxidative stress. iHAFCs-BMP9 underwent osteogenic differentiation, while iHNPCs differentiated towards chondrogenesis and secreted extracellular matrix. IHC results from both groups suggested that apart from macrophage aggregation due to foreign body reactions, no significant positive staining for CD68 or NF-κB was observed in the iHAFCs osteogenesis or iHNPCs chondrogenesis samples. NB, new bone; CM, chondrocyte matrix; PMI, peripheral macrophage infiltration; SC, seeded cells.

**
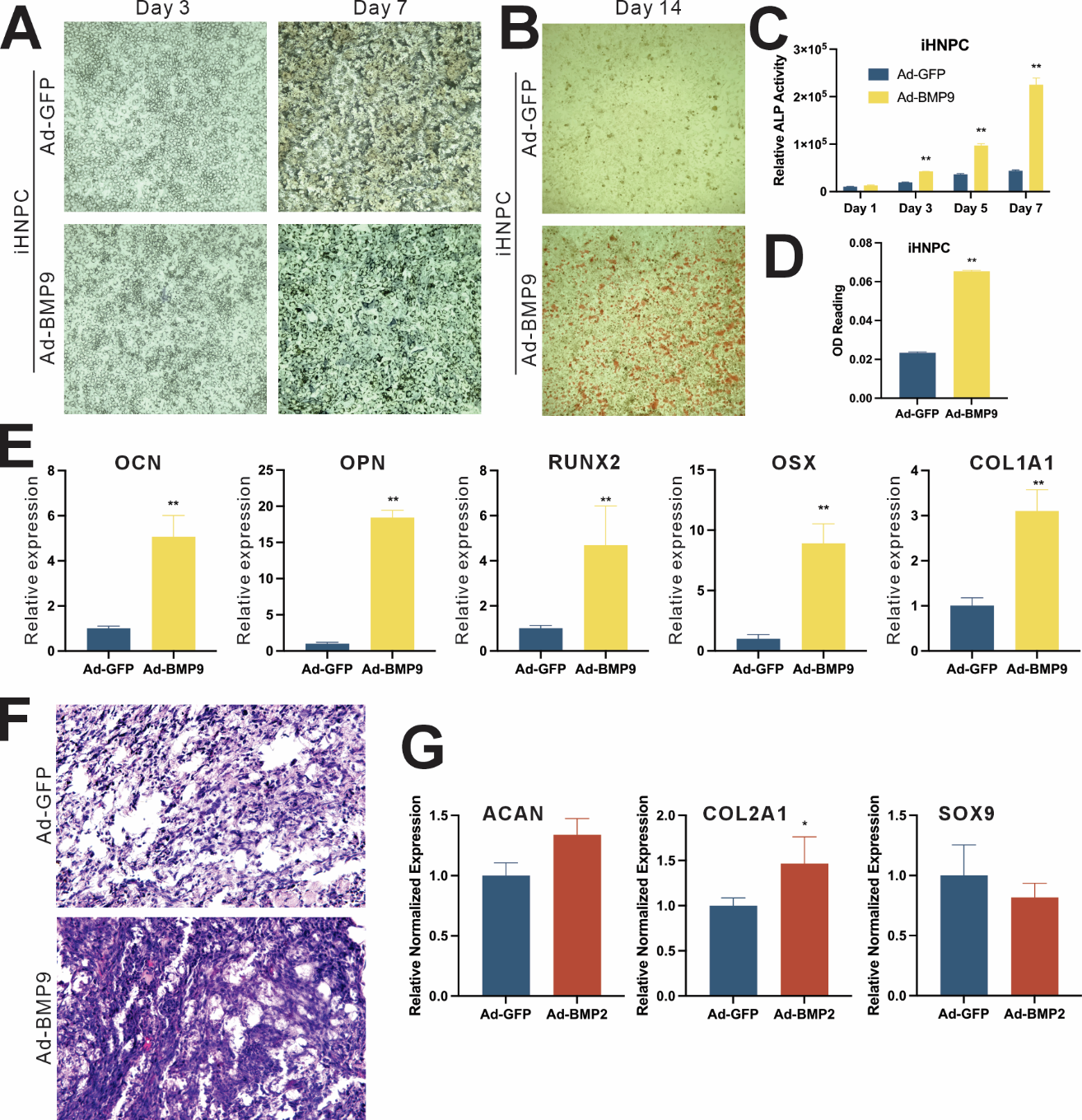
**

**Figure S3.** **Osteogenic potential of iHNPC and** **chondrogenic potential of iHAFC is limited. (A) to (F)** Osteogenic potential analysis of iHNPC. The experimental methods are the same as in Figure 3. Although some up-regulations of osteogenic related markers can be detected *in vitro* after BMP9 stimulation, iHNPC were not able to generate bone formation *in vivo*. **(G)** TqPCR analysis of chondrogenic markers of BMP2-induced iHAFC, indicating limited chondrogenic potential. “*”, p<0.05, compared that of GFP control.


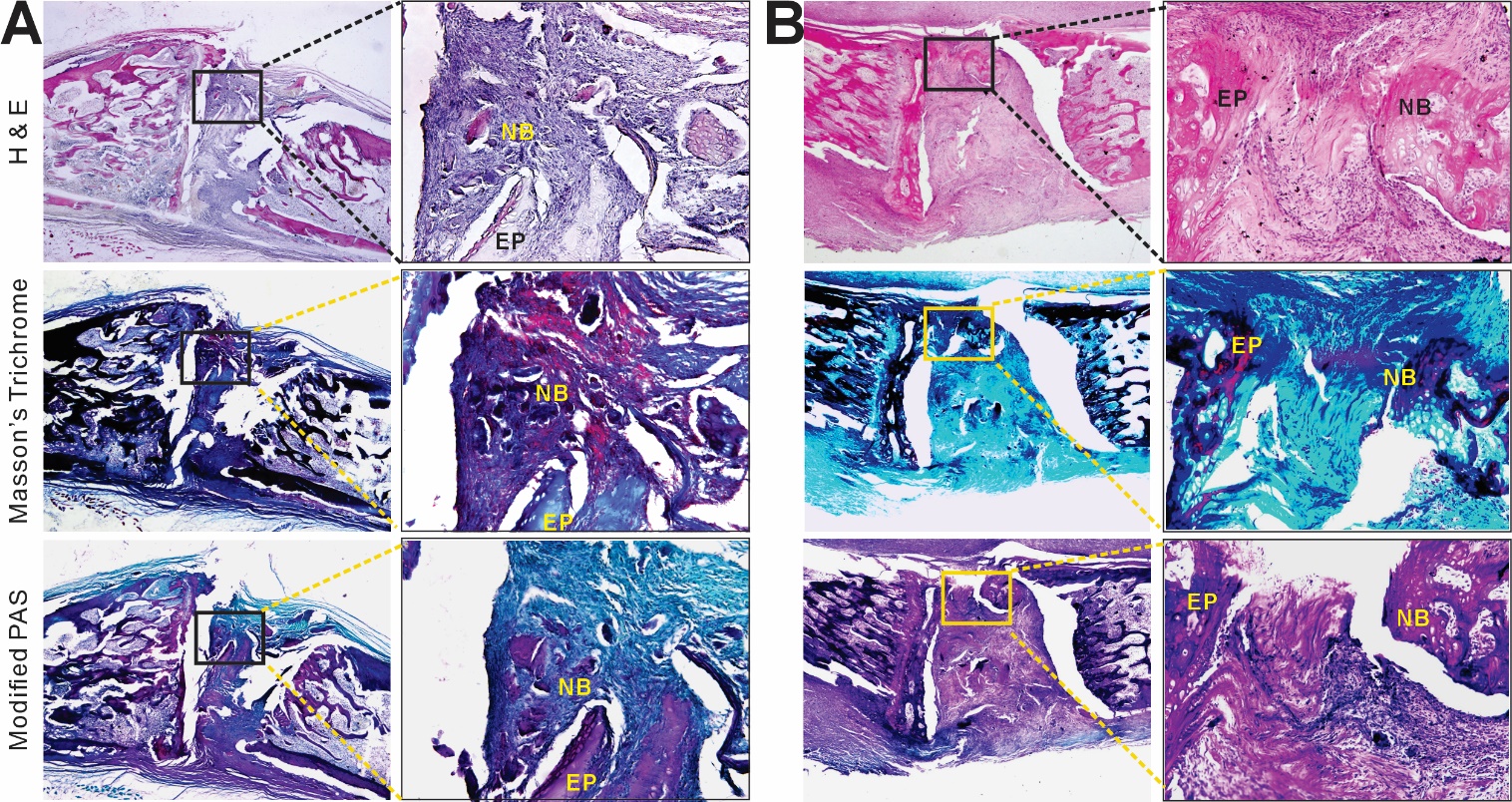


**Figure S4.** **More representative sections of *ex vivo* intervertebral fusion.** More retrieved samples from IVD fusion group (**A&B**) were analyzed. H & E staining, Masson’s trichrome staining, and modified PAS staining. Boxed regions are further magnified (right panel). Representative results are shown. NB, new bone; AF, annulus fibrosus; NP, nucleus pulposus; EP, endplate.
